# Swallowing and Liking of Vegetable-Enriched Bread Compared With Commercial Breads as Evaluated by Older Adults

**DOI:** 10.3389/fnut.2020.599737

**Published:** 2021-01-15

**Authors:** Isaac Amoah, Carolyn Cairncross, Elaine Rush

**Affiliations:** ^1^Faculty of Health and Environmental Sciences, Auckland University of Technology, Auckland, New Zealand; ^2^Riddet Institute, Massey University, Palmerston, New Zealand

**Keywords:** older adult, bread, swallowing, sensory evaluation, crumb texture

## Abstract

Characteristics of food that influence liking and ease-of-chewing and swallowing are not well-understood. Reformulation of bread to improve nutrient density may improve liking, ease-of-chewing and swallowing which could improve dietary intake particularly with aging. The study aimed to compare objectively and subjectively four breads of increasing nutrient density: $1 white (WB) and wheatmeal (WMB) commercial breads and two in-house formulations of vegetable-enriched breads (VB75 or VB100) which incorporated drum-dried pumpkin and sweet corn flours for physical, sensory and ease-of-chewing and swallowing properties. Each bread underwent instrumental texture analysis. The commercial and vegetable-enriched breads were not different by hardness or springiness but the vegetable breads were up to 25% less cohesive, less gummy and less chewy than the commercial breads. Questionnaires and Likert scale (150 mm) responses were completed by 50 physically active volunteers aged 50+ years. Overall liking of the VB75 and VB100 was rated 40% higher than the white and wheatmeal breads. Vegetable-enriched breads were rated as almost 50% easier to chew (mean ± SD; WB 70.53 ± 39.46 mm, WMB 77.68 ± 33.13 mm, VB75 104.78 ± 30.69 mm, VB100 107.58 ± 24.90 mm) and swallow (WB 70.29 ± 37.98 mm, WMB 77.53 ± 34.88 mm, VB75 104.63 ± 28.25 mm, VB100 104.90 ± 25.54 mm). Vegetable-enriched breads compared to white and wheatmeal breads were instrumentally and subjectively less gummy, cohesive and chewy than commercial breads and have the potential to both improve nutrition and “ease of swallowing” in older people. New areas of research should explore other underutilized vegetables for bread enrichment and their ability to aid swallowing and improve nutrition status.

## Introduction

Bread, a staple food in New Zealand (1), contributes 11% of total daily energy intake and older people are more likely to choose wholegrain bread (60%) than younger people (1). Therefore, the reformulation of bread to improve its nutrition for older people could be attractive to breadmakers and consumers of bread.

In New Zealand, the yearly sales of bread increased between the year to 19/06/2016 and the year to 18/06/2017 by 3.5% (from $462,491,400 to $479,077,700) (2). In the same trading period, non-white bread and specialty bread also increased in sales value by 3.3% (from $233,008,800 to $241,028,800) and 6.6% (from $67,855,300 to $72,670,200) respectively (2). In addition to the supply of energy and the macronutrients carbohydrate, protein and fat, breads may have health effects and can be called “functional breads” (3). Drum-dried pumpkin and sweet corn powders are two potential functional ingredients that could be utilized in bread formulation. Bioactive compounds including carotenoids (4) and essential micronutrients such as potassium are present in pumpkin (5) whilst sweet corn is a source of dietary fiber (6). Despite the potential benefits associated with functional bread consumption, the incorporation of functional ingredients in bread will impact on its physical properties including the textural properties of bread crumb and sensory attributes (7). Thus, there is the need to ensure a balance between sensory attributes and *nutritional* properties.

Particularly for older people, difficulties associated with chewing and swallowing *may* affect the ability to ingest certain foods including bread (8, 9) which may result in poor outcomes in their nutrition and health status (10). Many older people have discretionary funds which potentially increase their ability to buy palatable and nourishing bread which is easy to swallow. There is therefore the need to evaluate the physical properties, sensory attributes including “ease of swallowing” and demand of breads with the target market of older consumers.

The objective of this study was to evaluate the physical, sensory and swallowing attributes of two vegetable-enriched breads compared with controls, two commercially produced breads $1 white and wheatmeal breads, with 50 older physically active adults aged 50+. The hypotheses were that:

a. The vegetable-enriched breads will have softer crumb texture than the control breads possibly due to their fiber/pectin composition that has good moisture keeping properties.
b. The higher pumpkin flour content vegetable-enriched bread will be easier to swallow than the lower pumpkin flour content vegetable-enriched bread, and both will be easier to swallow than the control commercial breads.
c. The vegetable-enriched breads will be more liked and older adult consumers would be willing to eat them at home *possibly due to its potential softer crumb property*.

## Design and Methods

This experimental study involved the formulation with eight wholesome ingredients, including drum-dried pumpkin and sweet corn vegetable flour, of two vegetable–enriched breads to meet the Nothing Else™ criteria (11). The physical and subjective (human participant) measures were compared to controls of $1 commercial white and wheatmeal breads.

*The list of ingredients used for the vegetable bread formulation and places sourced is indicated below: strong white flour (Champion, Auckland), wholemeal flour (Champion, Auckland), whole flaxseed (Ceres Organic, Auckland), sprouted red wheat flour (Huckleberry, Auckland), pumpkin powder (Cedenco, Gisborne), sweet corn powder (Cedenco, Gisborne), yeast (Bakels, Auckland) and salt (Cerebos Skellerup, Auckland)*. In descending order by weight the ingredients were: strong white flour, wholemeal flour, *whole* flaxseed, sprouted wheat flour, pumpkin flour, sweet corn flour, fresh yeast and salt to produce two vegetable-enriched breads that differed only in the proportion of pumpkin powder; VB100 contained 25% more pumpkin powder (dry mix) than the VB75. The indirect method of bread-making was used for the bread development. A pre-ferment consisting of 225 g wholemeal flour, 150 g water, and 0.5 g instant yeast was developed for 2 min to tight dough consistency. The mixture was left covered for 12 h at 20°C to allow for fermentation. A 150 g portion of *whole flaxseed* was soaked in 180 g boiled water for 2 h at room temperature before placing in *a* chiller for 10 h. The final dough was prepared by mixing 450 g strong wheat flour, sprouted red wheat flour (115 g), pumpkin powder (75 g), sweet corn powder (20 g), salt (15 g), instant yeast (15 g) and water (600 g) for 8 min. The dough was developed for 6 min. The soaked *whole flaxseed* was added to the final mixture and mixed until incorporated. The dough was allowed to undergo bulk fermentation for one and half hours. The fermented dough was cut and shaped into logs and placed in tins and proved for an hour. The tins of dough were placed in a steam oven and baked at 215°C for 35 min. After baking, the VB75 breads were allowed to cool and packaged in transparent rubber packs. The same procedure was repeated for the VB100 breads except for the amount of pumpkin flour that was scaled up to 100 g. The recipe formulation for the vegetable breads used for the study is presented in Table 1. *The list of ingredients for the white bread included, in descending order by weight, wheat flour, water, baker's yeast, iodised salt, canola oil, acidity regulator (263), soy flour, emulsifier (481, 472e) and vitamin (folic acid). The wheatmeal bread contained wheat flour, water, wheatmeal flour, baker's yeast, vinegar, iodised salt, wheat gluten, acidity regulator (263), roasted barley malt flour, canola oil, soy flour, emulsifiers (481, 472e), and vitamin (folic acid)*.

**Table 1:** Recipe formulation for bread development.

**Ingredient**	**VB75**	**VB100**
Water	930 g	930 g
White strong flour	450 g	450 g
Wholemeal wheat flour	225 g	225 g
Flaxseed	150 g	150 g
Sprouted red wheat flour	115 g	115 g
Pumpkin powder	75 g	100 g
Sweetcorn powder	20 g	20 g
Instant yeast	5.5 g	5.5 g
Salt	15 g	15 g

*VB75-Bread enriched with 75 g pumpkin flour and VB100-Bread enriched with 100 g pumpkin flour*.

Nutritional information about the commercial breads was obtained from the nutrition information panel on the pack, for VB75 by proximate analysis at Asurequality, an Internationally Accredited New Zealand laboratory and for VB100 determined from the recipe and measured moisture loss using the software programme Foodworks 10 (Xyris, Brisbane) and the NZ food composition database (12) (Table 2). *The choice of the cheapest (NZ$1) commercial breads was premised on the fact that it is the most affordable bread in Countdown supermarket (50% of market share) in New Zealand*.

**Table 2:** Proximate composition of breads.

**Component**	^*^WB	^*^WMB	**^†^VB75**	**^††^VB100**
Moisture (%)	36.57	38.19	39.11	46
Protein (g/100 g)	8.5	8.8	*NA*	6.5
Dietary fiber (g/100 g)	2.7	4.6	7.2	6.5
Insoluble fiber (%)	*NA*	*NA*	5.5	*NA*
Soluble fiber (%)	*NA*	*NA*	1.7	*NA*
Fat (g/100 g)	1.6	1.7	*NA*	*NA*
Carbohydrate (g/100 g)	46.7	43.1	*NA*	36
Sodium (mg/100 g)	392	398	380	380
Potassium (mg/100 g)	*NA*	*NA*	300	277
Energy (kJ/100 g)	1020	982	*NA*	889
β-carotene (μg/100 g)	*NA*	*NA*	236.78	*NA*

* *As reported on the nutrition information panel*.

† *Analysis by Asurequality, an Internationally Accredited New Zealand laboratory*.

†† *Derived from recipe with the New Zealand Food Composition Tables (12). NA, not available; WB, white bread; WM, wheatmeal bread; VB75 and VB100, bread with 75 g and 100 g pumpkin substitution*.

### Physical Analyses: Texture Analysis

Texture profile analysis (TPA) measures of hardness, chewiness, cohesiveness, springiness, and resilience were determined using a (TA.XT.plus texture analyser, Stable Microsystems, Surrey, UK) with a 5 kg load cell. Crumb slices of 11.50 mm were 75% compressed. Parameters used include a pre-test speed of 5.00 mm/s, test speed of 1.00 mm/s, post-test speed of 5.00 mm/s, target mode-strain, time of 5.00 s and trigger force of 0.010 N. The resulting peak force was measured in grams. A minimum of five replicates from each of the sliced breads were averaged.

### Participants

The study was conducted according to the guidelines laid down in the Declaration of Helsinki, and all procedures involving human participants were approved by the Auckland University of Technology Ethics Committee (AUTEC) (New Zealand, 18/22).

*The number of participants required was calculated based on 108 previous studies of a wide range of food products including bread* (13). *When the meaningful difference over a 150 mm Likert scale was set at 20%, with an* α *of 0.05 and* β *of 0.10 the number of participants required was 29*.

Participants recruited were older (50+ years) and physically active people who were registered members of the never2old group (an exercise programme), and regularly attend the Sport and Fitness Center at the Auckland University of Technology, North Shore campus, Auckland, New Zealand. The participants were advised about this study by the leaders of the never2old programme and the advertisement was posted on the notice boards in the fitness center. The researcher gave a brief presentation to potential participants prior to a fitness session and information sheets were distributed *and the procedure explained*. Participants who responded to the invitation were recruited in a chronological manner. Participants were excluded if they were receiving drugs that would affect taste (e.g., chemotherapy) or were gluten intolerant or allergic to any of the ingredients. Inclusion criteria were: aged more than 50 years, consumed bread at least once a week, and had no known allergy or intolerance to gluten. After a one-on-one opportunity was provided for participants to ask any questions, an appointment was made for the testing.

*Fifty untrained participants (31 female, 19 male) consented to take part. One participant self-identified as Asian and the remainder as European. Twenty-six were aged 70–79 years (52%), twelve 80–89 years (24%), ten aged between 65–69 years (20%), and two were 50–59 years (4%)*.

### Liking, Acceptability, and Swallowing

*The widely-used questionnaire for liking and acceptability was as proscribed by Lawless and Heymann* (14). *Briefly the liking of each bread sample in relation to the sensory attributes (color, aroma, taste, texture, mouthfeel, overall liking, and willingness to eat at home) was rated on seven 150 mm unstructured visual analog scales with anchor points of extremely dislike on the left and extremely like on the right. Similarly five 150 mm scales were used to estimate “ease of swallowing” evaluation (extremely difficult to extremely easy) followed the sequence of the passage of food from the lips to the throat (ease of biting and getting into the mouth, ease of chew, ease of swallow, ease of throat movement, less stickiness in throat) and the number of chews before swallowing was counted by the participant. The swallowing questions were pre-tested for face validity and readability by colleagues*.

Participants attended the sensory evaluation sessions in a sensory room at the fitness center. *The procedure and the questionnaire was explained and demonstrated to the participants and opportunities to ask questions provided throughout. Participants then were asked to sign a consent form*. Each participant was seated at a table so that they could not see other participants. *On the table* were two slices each of each of the four different breads (one slice with crust and a second de-crusted slice of 11.50 mm square). *The sliced bread with intact crust weighed approximately 50 g*. The bread was served in an unrandomised (first 29 participants) and randomized (next 21 participants) order to the consumers on white plates identified with random three-digit numbers. For logistical reasons each portion of bread had been stored sealed and frozen and was allowed to defrost, sealed for a 1-h period. *Each bread was identified by a unique number*. Water was used to rinse the mouth between breads to minimize any residual effect between *breads. One questionnaire was provided* for each bread.

All the analyses were *undertaken with the* Statistical Package for Social Sciences (SPSS) version 24.0 software (IBM, New York). The results on physical properties and ease of swallowing of bread were subjected to one-way analysis of variance (ANOVA) *with bread as the grouping variable*. Means and 95% confidence interval *of differences in means are* reported. *Post hoc* Tukey's test was used to compare the mean values and establish significance differences at *p* < 0.05. *Although the initial unrandomisation order of presentation of the breads to the participants was a limitation, there were no significant differences between the attributes recorded by participants by randomized or not (p* > *0.05) and the data was pooled for the final analysis*. Sigma plot® software was used to visually establish the relationship between the objective and subjective perceptions associated with ease of swallowing.

## Results

Loaf weight, baking loss, loaf volume and specific volume of the VB75 and VB100 breads were not significantly different (Table 3). The VB75 and VB100 breads had substantially darker (>40% darker) crusts than the WB and WMB. The lightness, redness, and yellowness colors of the crusts of the VB75 and VB100 breads were not different.

**Table 3:** Physical and textural attributes of the four breads.

**Characteristics**	**N**	**Bread samples**					
		**WB**	**WMB**	**VB75**	**VB100**	***P*-value**
**Physical, textural attributes**						
Loaf weight (g)	4			420.0 ± 1.6^a^	421.8 ± 2.5^a^	0.556
Baking loss (%)	4			10.4 ± 0.3^a^	10.1 ± 0.5^a^	0.556
Loaf volume (ml)	4			1,027.7 ± 25.1^a^	1,047.3 ± 11.9^a^	0.381
Specific loaf volume (mL/g)	4			2.5 ± 0.1^a^	2.5 ± 0.0^a^	0.498
**Loaf crust color**						
L*	7	57.1 ± 6.5^b^	53.26 ± 1.7^b^	33.2 ± 1.0^a^	35.56 ± 4.1^a^	<0.0001
a*	7	17.5 ± 1.3^b^	17.4 ± 1.1^b^	14.3 ± 0.9^a^	15.4 ± 0.7^a^	<0.0001
b*	7	35.5 ± 2.9^b^	33.0 ± 1.3^b^	19.2 ± 1.2^a^	22.1 ± 2.6^a^	<0.0001
**Loaf crumb color**						
L*	10	83.7 ± 2.8^c^	77.8 ± 2.5^b^	55.0 ± 4.5^a^	55.4 ± 3.6^a^	<0.0001
a*	10	−0.1 ± 0.2^a^	2.0 ± 0.6^b^	3.2 ± 0.8^c^	4.3 ± 1.4^d^	<0.0001
b*	10	10.3 ± 1.0^a^	15.3 ± 1.8^b^	36.1 ± 2.5^c^	40.0 ± 1.8^d^	<0.0001
Hardness (g)	5–9	8.49 ± 1.74^a^	8.51 ± 1.00^a^	8.68 ± 2.23^a^	10.06 ± 1.09^a^	0.131
Resilience (%)	5–9	28.78 ± 3.14^b^	33.41 ± 3.22^c^	27.06 ± 3.23^b^	20.58 ± 2.65^a^	<0.0001
Cohesion	5–9	0.74 ± 0.05^c^	0.80 ± 0.04^c^	0.60 ± 0.06^b^	0.52 ± 0.02^a^	<0.0001
Springiness (%)	5–9	84.74 ± 17.87^a^	91.51 ± 1.84^a^	87.55 ± 4.46^a^	82.36 ± 5.62^a^	0.170
Gumminess	5–9	6.21 ± 1.14^a, b^	6.80 ± 0.80^b^	5.19 ± 1.36^a^	5.23 ± 0.51^a^	0.006
Chewiness	5–9	5.41 ± 1.81^a, b^	6.23 ± 0.79^b^	4.59 ± 1.33^a^	4.30 ± 0.43^a^	0.007

Data is expressed as mean ± standard deviation. Values with different superscript in a row are significantly different (p <0.05). WB, white bread; WMB, wheatmeal bread, VB75 and VB100, breads with 75 g and 100 g pumpkin substitution. Means with different superscripts in a column are significantly different (p < 0.05). N= Number of replicates. L* indicates the lightness value, a* indicates the degree of redness, and b* indicates the degree of yellowness. A higher the L*, a*, and b* value means a higher degree of lightness, redness, and yellowness of the bread, respectively. A higher hardness, resilience, cohesion, gumminess, and chewiness value indicates a higher degree of bread crumb hardness, resilient, cohesion, springinessy, gumminess, and chewiness of the bread crumb, respectively, following the double compression measurements of using the texture analyser probe.

Physical measures of hardness and springiness among the four breads were not significantly different. The WMB was the most resilient to compression and had higher cohesion than the two vegetable-enriched breads but not the WB (Table 3). Resilience and cohesion of VB75 were higher than VB100 but springiness, gumminess and chewiness were not different between the two vegetable-enriched breads. Objective chewiness was higher for the WMB compared with both the VB75 (1.64 units, 95% CI 0.42, 3.25 *p* = 0.043) and VB100 (1.93 units, 95% CI 0.49, 3.37, *p* = 0.006) but not the WB.

Evaluated by the participants, the VB75 and VB100 breads were liked almost twice as much as the WB and WMB for all the sensory attributes assessed. The participants also stated that they were willing to eat the VB75 and VB100 breads at home (Table 4). There were no differences between the WMB and the WB for the liking attributes except for the color *of* the WMB *which was liked more* than the WB. Both the VB75 and VB100 recorded scores almost twice those of the WB and WMB for willingness to eat at home (Table 4).

**Table 4:** Sensory liking and swallowing perceptions of the four breads by participants (*n* = 50).

**Characteristics**	**Bread samples**					
	**WB**	**WMB**	**VB75**	**VB100**	***P*-value**
**Sensory attributes: liking (mm/150 mm)**					
Color	54.24 ± 37.23^a^	80.65 ± 34.42^b^	85.74 ± 33.42^b^	87.54 ± 37.66^b^	<0.0001
Aroma	73.12 ± 31.06^a^	76.16 ± 27.49^a^	95.17 ± 27.30^b^	92.16 ± 34.12^b^	<0.0001
Taste	52.77 ± 31.09^a^	60.22 ± 29.27^a^	93.42 ± 30.00^b^	94.10 ± 34.27^b^	<0.0001
Texture	46.70 ± 30.81^a^	60.71 ± 30.93^a^	96.69 ± 28.78^b^	97.60 ± 31.89^b^	<0.0001
Mouthfeel	47.46 ± 30.26^a^	54.34 ± 27.57^a^	94.36 ± 27.57^b^	91.09 ± 36.44^b^	<0.0001
Overall liking	40.85 ± 31.59^a^	52.43 ± 32.80^a^	92.03 ± 34.24^b^	93.58 ± 35.52^b^	<0.0001
Willing to eat at home	30.19 ± 33.40^a^	47.64 ± 34.24^a^	84.67 ± 42.89^b^	86.47 ± 42.21^b^	<0.0001
**Swallowing evaluation/150 mm**					
Ease of biting and getting into the mouth	98.31 ± 31.79^a^	99.56 ± 26.53^a, b^	113.29 ± 28.32^b^	111.34 ± 26.47^a, b^	0.012
Ease of chew	70.53 ± 39.46^a^	77.68 ± 33.13^a^	104.78 ± 30.69^b^	107.58 ± 24.90^b^	<0.0001
Ease of swallow	70.29 ± 37.98^a^	77.53 ± 34.88^a^	104.63 ± 28.25^b^	104.90 ± 25.54^b^	<0.0001
Ease of throat movement	77.33 ± 39.75^a^	78.18 ± 34.78^a^	108.61 ± 27.53^b^	110.41 ± 23.03^b^	<0.0001
Less stickiness in throat	72.33 ± 45.45^a^	75.94 ± 39.64^a^	107.62 ± 33.46^b^	111.48 ± 29.18^b^	<0.0001
Number of chews before swallowing	19	21	18	19

*Data is expressed as mean ± standard deviation. Values with different superscript in a row are significantly different (p < 0.05). Scoring is on a Likert scale 0–150 mm with 0 mm as the least and 150 mm as the most acceptable. WB, white bread; WM, wheatmeal bread; VB75 and VB100, breads with 75 g and 100 g pumpkin substitution. Means with different superscripts in a column are significantly different (p < 0.05)*.

WB was perceived as more difficult to bite and get into the mouth, chew, swallow, move through the throat compared with the other breads (Table 4) and also that it stuck in the throat more during swallowing. The VB100 was perceived as the easiest to chew and swallow and moved more easily through the throat with less throat stickiness. The swallowing attributes of VB75 bread were, however, not significantly different from the VB100 in terms of ease of bite and getting into the mouth (*p* = 0.99), ease of chew (*p* = 0.97), ease of swallow (*p* = 1.00), ease of throat movement (*p* = 0.99) and less stickiness in throat (*p* = 0.96). The overall liking of all the breads was strongly correlated with the ease of swallowing: VB75, *r* = 0.597, 95% (0.3820.751); VB100, *r* = 0.665, 95% (0.474–0.796); WB, *r* = 0.422, 95% (0.163–0.627); and WMB, *r* = 0.475, 95% (0.227–0.665).

**Figure 1:**
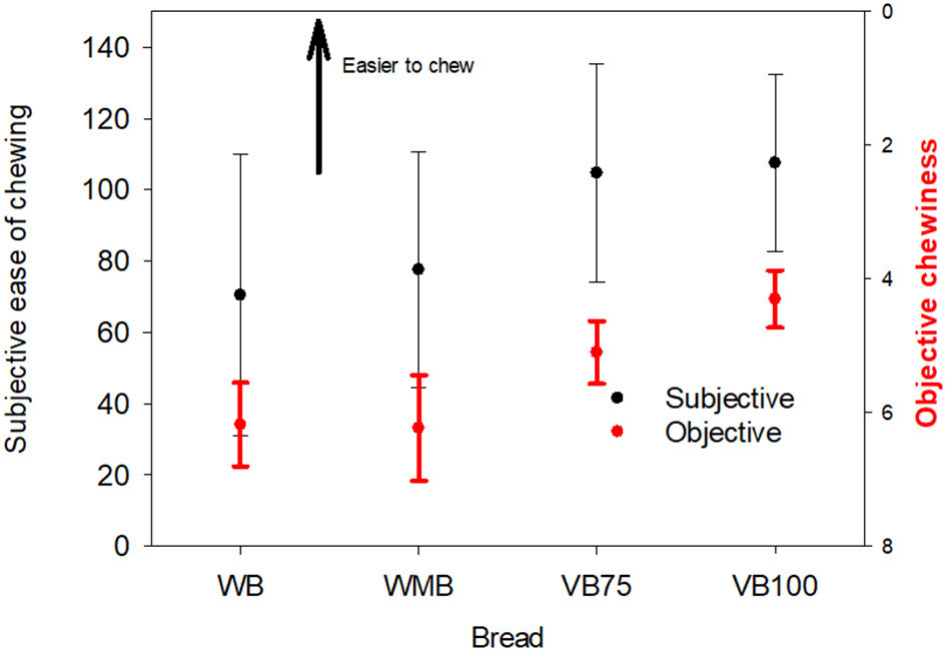
Relationship between subject-rated ease of chewing and objective chewiness of bread samples. Means and 95% CI. WB, white bread; WM, wheatmeal bread; VB75 and VB100, bread with 75 g and 100 g pumpkin substitution.

Participants reported the two commercial breads as less easy to chew, to get in the mouth and swallow than the vegetable-enriched breads (Figure 1 and Table 4) which is in the same direction as objective measures which discriminated the commercial breads as chewier, more cohesive, and gummier than the vegetable breads.

## Discussion

Vegetable-enriched breads were successfully formulated and produced. This study has shown that older participants substantially preferred the taste of the VB75 and VB100 breads over the commercial breads and would be willing to eat the vegetable-enriched breads at home. They also subjectively ranked the vegetable-enriched breads as easier to chew and swallow than the commercial white and wheatmeal breads. This confirmed the hypotheses that the higher pumpkin flour concentration vegetable-enriched bread (VB100) will be easier to swallow than the lower pumpkin concentration vegetable-enriched bread (VB75), and both will be easier to swallow than the commercial control breads (WB and WMB). The objective measures of chewiness and cohesion were the only physical measures able to differentiate between the commercial breads and the vegetable-enriched breads in the same direction as the participants' perceived ease of chewing. A novel ease-of-swallowing “solid food” questionnaire was trialed, found to be understood by and acceptable to participants, and showed subjective discrimination of the ease of stages of ingestion among breads that the objective measures did not.

Explanations for the easier chewing and swallowing of the vegetable-enriched breads related to an easier formation and passage of the bread bolus. This may be attributed to pumpkin powder containing pectin (15) which is rich in hydrophilic fibers (16), increasing the bulk of the bread in the mouth and a potential increased saliva stimulation. Saliva promotes the formation of cohesive network between the bread bolus in the mouth, consequently aiding swallowing (17). *Soaking of unground flaxseed results in the formation of a viscous mucilage which has emulsifying properties* (18). *Additionally, flaxseed has a fat content of 42.2 g/100 g* (12). *Fats reduce the adhesiveness of the food bolus formed in the mouth and thus putatively, if well chewed, could partially contribute to the ease of swallowing of the bread bolus. Gluten from the wheat flour, provides viscoelasticity to the dough and helps with the retention of carbon dioxide gas produced after fermentation* (19, 20). *The addition of the vegetable powders to the wheat flour dilutes the gluten which may have the effect of softening the bread making it easier to chew and swallow. In addition, flaxseed by mass is* ~*25% fiber, 70% insoluble and contributed substantially (30%) to the total fiber in the bread* (12). *It is therefore likely that flaxseed is the ingredient that may have augmented many of the outcomes, and in particular texture*.

Increased salivation *may be* stimulated by color of bread (21) *and can impact* on consumers' choices. Bread with white crumb is perceived to be *less healthy*, particularly amongst older people, as indicated anecdotally by the participants. The yellow color of the VB probably created an impression of healthiness in the minds of the participants, consequently resulting in the higher liking of the VB. Kraus (22) reported that drivers for consumers' liking of food are dependent on healthiness and naturalness.

The vegetable-enriched breads were more liked for their taste compared to the commercial WB and WMB breads. A plausible reason could be the action of saliva as a medium for the dilution of taste compounds including sugar and salt (23) from the vegetable-enriched breads. The diluted compounds are subsequently conveyed to taste receptors on the surface of the tongue (24, 25) and are perceived to be appealing by the participants.

An explanation for the liking of the aroma of the vegetable-enriched breads could be in relation to the role of saliva. Mosca and Chen (24) postulated that saliva increases the availability of aroma compounds from food *as the* food *is* broken into smaller particle sizes during chewing. The released aroma compounds attach themselves to receptors in the mouth while some diffuse into cavities of the nose leading to flavor perception which in turn results in increased salivation (26). Aroma released from bread also impacts on the release of saliva. Studies on the flavor volatiles available in sweet corn revealed the presence of aroma compounds including dimethyl sulfide, 2-acetyl-1-pyrroline and 2-acetyl-2-thiazoline (27). Interestingly, 2-acetyl-1-pyrroline, which is an essential flavor compound produced from Maillard reaction in sweet corn, is noted for its appealing flavor in bread (28). 2-acetyl-2-thiazoline, on the other hand, is found to generate a roasty popcorn-like favor in bread (28). Other compounds including hydrogen sulfide, methanethiol, acetaldehyde, ethanol, ethanethiol, dimethyl sulfide impact the aroma of thermally processed sweet corn (29). The presence of these compounds in addition to the Maillard reaction that takes place during the baking process possibly resulted in the generation of appealing aromatic compounds in the vegetable-enriched breads. This could be attributed to the degradation and modification of the cell walls of the vegetable ingredients which may result in an aroma favorable to the consumers (30). A strong positive association between the liking of food and its aroma composition has been reported (31) thus the higher liking score recorded for the vegetable-enriched breads by the participants may be justified. Additionally, the pre-fermentation of the wholemeal flour for 12 h using yeast possibly improved the textural properties and promoted the release of certain volatile and aromatic compounds in the vegetable-enriched breads.

*It is also worth highlighting that during bread chewing, the texture of the bread matrix impacts on the release of aroma compounds* (32). *In the present study, participants had a favorable perception of the crumb texture of the vegetable-enriched breads leading to an appealing mouthfeel. Consequently, salivation of the bread bolus increased, resulting in the ease of bread swallowing*. The vegetable-enriched breads were reported to be easier to chew. Chewing is a mechanical process that stimulates the release of saliva (33) consequently promoting increased bread bolus lubrication (17). This possibly resulted in easier swallowing and movement of the bread bolus through the throat (24).

*With aging the mass of the swallowing muscles declines* (34) *and swallowing may be less efficient. Thus, for older people*, the vegetable-enriched Nothing Else™ breads, if consumed, could be favorable for their overall nutritional intake.

The older participants subjectively found white bread more difficult to chew and swallow though there was no difference between its crumb hardness and that of the vegetable-enriched breads as evaluated objectively. This was expected, as white bread is formulated from refined white flour, poor in fiber and contains emulsifiers which improves bread crumb textural attributes (35, 36). Consequently, after bread chewing, bolus swallowing may get impaired as it clogs in the throat, and this was confirmed anecdotally by the participants. The commercial sold white and wheatmeal breads contained emulsifiers 481 (sodium oleyl lactylate, sodium stearoyl lactylate, and sodium lactylate) and 472e (diacetyltartaric and fatty acid esters of glycerol) (37) to improve the bread crumb textural properties and to cause the bread to feel softer in the plastic bag and therefore appear fresher (36). This likely contributed to the lower hardness of the white breads when measured with the texture analyser, as the use of emulsifiers has been reported to improve the textural properties of bread (35, 36). The use of food additives including hydrocolloids to enhance the properties of bread, especially in commercially sold bread, is a common and accepted practice. However, particularly amongst the health-conscious older population, consumers are avoiding food products with food additive enhancement as they see them as “unnatural” (38).

There is potentially an adverse effect of the higher moisture content in the vegetable-enriched bread VB100 as higher water activity leads to early spoilage by microbiological organisms, especially mold (39–41). However, in a review paper recently published (42), we posited that functional breads tend to have a longer shelf life than white breads due to antimicrobial and antioxidant bioactivity compounds present in the functional ingredients which impair the growth rate of mold and the oxidation of lipids and fats in the bread matrix.

## Strengths and Limitations

*This analysis was limited by the relatively small number of breads to compare* (4) *and the convenience sample of healthy older adults enrolled in a fitness programme. The participants were not asked if they had any difficulties swallowing food or if they required water to help them swallow. In addition, the unavoidable lack of blinding to, for example, the color and appearance of the breads may have created bias in the assessment of ease of swallowing. The initial lack of randomisation of order of presentation of the breads may also have created bias but there appeared to be no difference between the randomized versus unrandomised results so they were pooled. Additional experiments and measurements would provide more information and helped identify ingredients responsible for the characteristics but also would increase participant burden, reduce compliance and lengthen the swallowing questionnaire. More food chemistry analyses of the breads would provide the information missing in* *Table 2**. Future comparison of swallowing characteristics of foods reformulated for a better nutritional profile and easier swallowing would aid both food manufacturers, marketeers and consumers*.

## Conclusion

The enrichment of bread with pumpkin and sweet corn and the pre-ferment dough preparation was apparently associated with overall liking and improved ease of chewing and swallowing. Future interdisciplinary research should focus on understanding the microstructure of foods and subjective ease of swallowing. New areas of research should explore other underutilized vegetables for bread enrichment and their ability to aid swallowing and improve nutrition status, and the utility of a subjective swallowability index for foods for consumers.

## Data Availability Statement

The anonymised raw data supporting the conclusions of this article will be made available by the authors, without undue reservation.

## Ethics Statement

The studies involving human participants were reviewed and approved by Auckland University of Technology Ethics Committee (AUTEC) (New Zealand, 18/22). The patients/participants provided their written informed consent to participate in this study.

## Author Contributions

IA formulated the research question, designed the study, carried it out, analyzed the data and prepared the first draft. CC provided logistic support and supervision. ER obtained the funding for IA, oversaw the research process from conception, analyzed the data and provided logistic support and supervision. All authors provided critical revision of the article.

## Conflict of Interest

The authors declare that the research was conducted in the absence of any commercial or financial relationships that could be construed as a potential conflict of interest.
